# Tetradiketone macrocycle for divalent aluminium ion batteries

**DOI:** 10.1038/s41467-021-22633-y

**Published:** 2021-04-22

**Authors:** Dong-Joo Yoo, Martin Heeney, Florian Glöcklhofer, Jang Wook Choi

**Affiliations:** 1 School of Chemical and Biological Engineering and Institute of Chemical Processes, Seoul National University, Gwanak-Gu, Seoul Republic of Korea; 2 Department of Chemistry and Centre for Processable Electronics, Imperial College London, London, UK; 3 Department of Materials Science and Engineering, Seoul National University, Gwanak-Gu, Seoul Republic of Korea

**Keywords:** Batteries, Batteries, Batteries

## Abstract

Contrary to early motivation, the majority of aluminium ion batteries developed to date do not utilise multivalent ion storage; rather, these batteries rely on monovalent complex ions for their main redox reaction. This limitation is somewhat frustrating because the innate advantages of metallic aluminium such as its low cost and high air stability cannot be fully taken advantage of. Here, we report a tetradiketone macrocycle as an aluminium ion battery cathode material that reversibly reacts with divalent (AlCl^2+^) ions and consequently achieves a high specific capacity of 350 mAh g^−1^ along with a lifetime of 8000 cycles. The preferred storage of divalent ions over their competing monovalent counterparts can be explained by the relatively unstable discharge state when using monovalent AlCl_2_^+^ ions, which exert a moderate resonance effect to stabilise the structure. This study opens an avenue to realise truly multivalent aluminium ion batteries based on organic active materials, by tuning the relative stability of discharged states with carrier ions of different valence states.

## Abstract (web summary)

Aluminium ion batteries have been developed based on the storage of monovalent complex ions, impairing their original motivation of storing multivalent ions. Here, the authors demonstrate the divalent ion storage of tetradiketone macrocycles by tuning the relative stability of discharged states.

## Introduction

State-of-the-art lithium-ion batteries (LIBs) are predominantly used as power sources for a variety of mobile electronic devices and electric vehicles. Although LIBs have made remarkable progress in terms of various aspects of battery performance, attention to so-called ‘beyond LIBs’ is incessantly growing, mainly because of concerns related to the unpredictable supply of raw materials employed in LIBs^1,2^. In particular, the uneven distribution and fluctuating price of lithium and transition metal precursors reflect these shortcomings^3,4^. Moreover, the innate safety risk associated with organic electrolyte-based LIBs has driven the on-going search for alternative safe battery systems. Among these candidates, aluminium (Al)-based batteries have recently received considerable attention because Al is the third most abundant element in the Earth’s crust and, in its metallic form, it can deliver a high theoretical capacity of 2980 mAh g^−1^ or 8506 mAh cm^−^^3^ by taking advantage of the trivalent charge state^5–7^. Metallic Al is also stable upon exposure to air and moisture, unlike its Li and Na metal counterparts. Nonetheless, the development of aluminium ion batteries (AIBs) has lagged behind at the research stage. One of the main hurdles to overcome is to develop cathode materials capable of storing multivalent Al-containing carrier ions. In practice, finding such materials with sufficiently high ionic conductivity is a non-trivial matter, because the multi-valency of Al or Al-complex ions induces strong Coulombic interaction with anionic frameworks^8,9^.

Ironically, most cathode materials identified to date in AIBs operate by storing monovalent Al-complex ions, such as AlCl_4_^−^. These materials include carbon materials^10–13^, metal sulfides^14–16^ and metal selenides^17,18^. In the case of carbon materials, ever since the original report^19^ by Lin et al., who revealed the reversible storage of AlCl_4_^−^ ions in graphite, various graphene-based materials were explored. However, this reaction, which exploits AlCl_4_^−^ ions from the electrolyte, is unfavourable from an energy density consideration because the amount of electrolyte becomes a factor limiting the energy density. On the other hand, certain metal oxides^20–22^ and sulfides^23–25^ exhibited reversible storage of trivalent Al ions (Al^3+^). However, the strong Coulombic interaction between Al^3+^ and the hosts makes facile ion diffusion difficult at reasonably high current densities. Rather, Al^3+^ ions could unwantedly transform oxides and sulfides to Al_2_O_3_ and Al_2_S_3_ at the surface^26,27^. Notably, Chevrel-phase molybdenum sulfide (Mo_6_S_8_) with exceptionally large ionic channels is the only host material that has been proven to reversibly store Al^3+^ ions. However, the available specific capacity was only 100 mAh g^−1^ with a low operating voltage of 0.5 V even at the high temperature of 50 °C^28,29^.

The choice of materials for AIB cathodes was recently expanded to a family of organic molecules^30–34^ because the availability of sufficient intermolecular space as a result of the weak Van der Waals intermolecular interactions can be utilised for the diffusion and storage of the bulky aluminium complex carrier ions. These internal structures are also beneficial for releasing the strain generated during repeated insertion and extraction of the bulky complex ions, thereby achieving long-term cyclability. Despite these structural advantages, organic molecules are still not able to utilise the multi-valency of the Al ion for storage in their intermolecular space, thus they are adversely affected by the same limitation as the aforementioned series of inorganic materials.

Herein, we report a tetradiketone (TDK) macrocycle as a high-capacity cathode material for divalent AIBs. Particularly, the design we introduce exploits the radical destabilisation effect in the active molecule to preferentially induce divalent ion storage. A combination of density functional theory (DFT) calculations and experimental analyses reveal that adjacent carbonyl groups located at each corner of the TDK reversibly bind with a divalent AlCl^2+^ ion upon reduction, delivering an exceptionally high specific capacity of 350 mAh g^−1^ as an AIB cathode. Moreover, TDK exhibited excellent cyclability of 78% retention after 8000 cycles. This study offers useful insights into the way in which active molecules can be designed to activate their capability of storing divalent aluminium ions and thus take due advantage of multivalent batteries.

## Results

### Design and properties of TDK macrocycle

The TDK macrocycle was synthesised (Supplementary Fig. 1) by adapting and combining the procedures developed by Miljanić and Bunz et al.^35,36^ to improve the purity of the target compound and double the overall yield. In the first step of the synthesis, cyclotetrabenzoin was produced as an intermediate, which was isolated by filtration for use in the next step, that is, its oxidation to TDK in concentrated HNO_3_. The crude product was obtained in solid form by filtration. Subsequent Soxhlet extraction with chloroform selectively dissolved TDK and separated it from insoluble side products. The structure and purity of the final product was confirmed by a combination of nuclear magnetic resonance (NMR), Fourier-transform infra-red (FT-IR) and thermogravimetric analysis (TGA) (Supplementary Figs. 2 and 3).

**Fig. 1:**
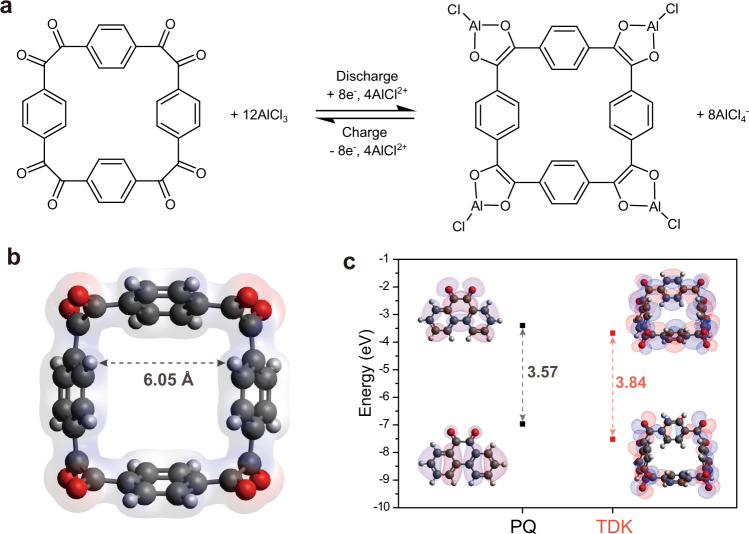
Design and properties of the tetradiketone (TDK) macrocycle. **a** Illustration of electrochemical redox mechanism of TDK. **b** Optimised structure and electron density of TDK in its pristine state. **c** HOMO-LUMO energy levels of the optimised structures of phenanthrenequinone (PQ) and TDK.

The reaction scheme of TDK with AlCl_3_ is presented in Fig. 1a. From the perspective of its chemical structure, TDK can be reduced (or discharged) by accepting as many as 8 e^−^ in such a way that the adjacent carbonyl groups at each corner form a chelate with one AlCl^2+^ ion. The reverse reaction occurs during oxidation (or charging). The benzene rings in TDK are not equiplanar with the macrocyclic plane owing to their rotational freedom, and a large void of 6.05 Å is present in the centre of the macrocycle (Fig. 1b). This void could possibly facilitate efficient transport of the charge carrier ions. Note that this molecular structure of TDK is in contrast with that of phenanthrenequinone (PQ), another representative molecule that would be suitable as an AIB cathode material, in that PQ has a planar structure comprising a series of conjugated bonds without rotational freedom (see structure in Fig. 1c, inset). The DFT calculations revealed that, despite the disconnected conjugated bonds in the macrocycle, the energy difference between the highest occupied molecular orbital (HOMO) and lowest unoccupied molecular orbital (LUMO) of TDK is 3.84 eV, which closely approximates the 3.57 eV of PQ (Fig. 1c).

### Reaction mechanism of TDK with divalent aluminium complex ions

**Fig. 2:**
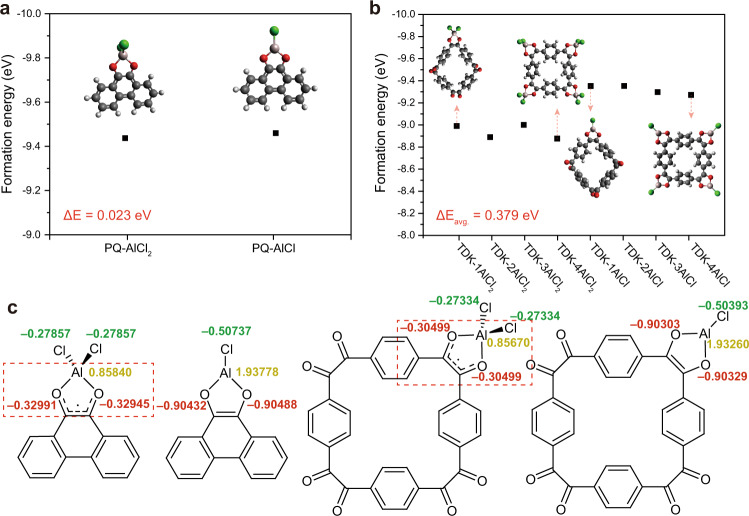
Thermodynamic stability of PQ and TDK when bound with monovalent (AlCl_2_^+^) or divalent (AlCl^2+^) carrier ions. **a**, **b** Formation energies of **a** PQ and **b** TDK when bound with AlCl_2_^+^ or AlCl^2+^ complex ions. The four different states correspond to the cases when different numbers of AlCl_2_^+^ or AlCl^2+^ ions are bound. The optimised molecular structures of selected TDK-*n*AlCl_2_ or TDK-*n*AlCl combinations are presented. **c** Atomic charge analysis of PQ and TDK when bonded to a single AlCl_2_^+^ or AlCl^2+^ ion.

To elucidate the reaction mechanism of TDK in comparison with PQ, we conducted DFT calculations to estimate the formation energies of both molecules when each molecule binds with a monovalent (AlCl_2_^+^) or divalent (AlCl^2+^) complex ion. All the structures in Fig. 2a, b are structurally optimised states (details of the calculation appear in the Method section). As the formation energy refers to thermodynamic stability, the formation energies of the discharged states can uncover the preferred charge carriers for the electrochemical reaction in electrolytes in which Al_2_Cl_7_^−^ can yield various forms of aluminium complex ions such as AlCl_4_^−^, AlCl_2_^+^, AlCl^2+^ and Al^3+^. In the case of PQ and TDK, the adjacent carbonyl groups possibly chelate AlCl_2_^+^ or AlCl^2+^ ions upon reduction. Therefore, the energy difference between the states bonded with these two carrier ions can be taken as a descriptor for indicating the major carrier ion.

In the case of PQ, only one possible stable configuration exists upon binding with each carrier ion (Fig. 2a), denoted as PQ-AlCl_2_ and PQ-AlCl, respectively. The formation energies of PQ-AlCl_2_ and PQ-AlCl were −9.436 and −9.459 eV, respectively, implying that PQ-AlCl is more thermodynamically stable. However, considering that the energy difference between the two complex states is merely 0.023 eV, the PQ-AlCl_2_ form cannot be ruled out in reality. Taking ionic diffusion into account, binding with AlCl_2_^+^ ions could be overall preferred, as was indeed experimentally verified^32,37^. On the other hand, in the case of TDK, a total of eight possible states can be postulated: four with AlCl_2_^+^ ions and the other four with AlCl^2+^ ions. To note, although there are two possible configurations for TDK-2AlCl_2_ and TDK-2AlCl in which the carrier ions are located in the adjacent and opposite positions, the configurations with lower formation energy are presented. The average formation energies of TDK-*n*AlCl_2_ and TDK-*n*AlCl were calculated to be −8.939 and −9.318 eV, respectively, implying that binding with AlCl^2+^ ions is thermodynamically more favourable. Furthermore, the energy difference between TDK-*n*AlCl_2_ and TDK-*n*AlCl was found to be 0.379 eV, which is beyond the range of the overpotential in typical electrochemical measurements. This suggests that TDK prefers divalent over monovalent ion storage.

Importantly, the preferential binding of TDK with the AlCl^2+^ ion can be understood in relation to its relative instability upon binding with the AlCl_2_^+^ ion when compared with PQ. It is worth noting that a quinone molecule normally generates a radical upon reduction with one electron (and binding with a monovalent ion), and the radical is stabilised by the delocalisation with the adjacent aromatic rings. From the viewpoint of the chemical structure, TDK has a lower benzene ring-to-carbonyl group ratio of 0.5 compared to 1.0 of PQ, resulting in a less pronounced stabilisation of radicals by the resonance effect. This equally means that upon the formation of a radical, TDK can form a smaller number of resonance structures than PQ. This resonance effect was also revealed by atomic charge analysis as shown in Fig. 2c. In the pristine state, the oxygen atoms of PQ and TDK have a similar atomic charge of −0.5502 and −0.5429 on average, respectively (Supplementary Fig. 4). When an AlCl_2_^+^ ion is bound, the oxygen atoms of PQ each have an atomic charge of −0.3297, whereas that of TDK is clearly smaller at −0.3049, indicating weaker binding with the AlCl_2_^+^ ion. This weaker binding of TDK with AlCl_2_^+^ is once again associated with the less prominent stabilisation of radicals by the delocalisation. By contrast, when an AlCl^2+^ ion is bound, the oxygen atoms of PQ and TDK have a similar atomic charge of −0.9045 and −0.9031, respectively, indicating that the binding strength with the AlCl^2+^ ion is similar for both molecules. Therefore, the difference in binding strength between the AlCl_2_^+^ and AlCl^2+^ ions is greater for TDK, and serves as the origin of its preferential binding with the AlCl^2+^ ion.

### Ex situ characterisation of TDK in the discharged state

**Fig. 3:**
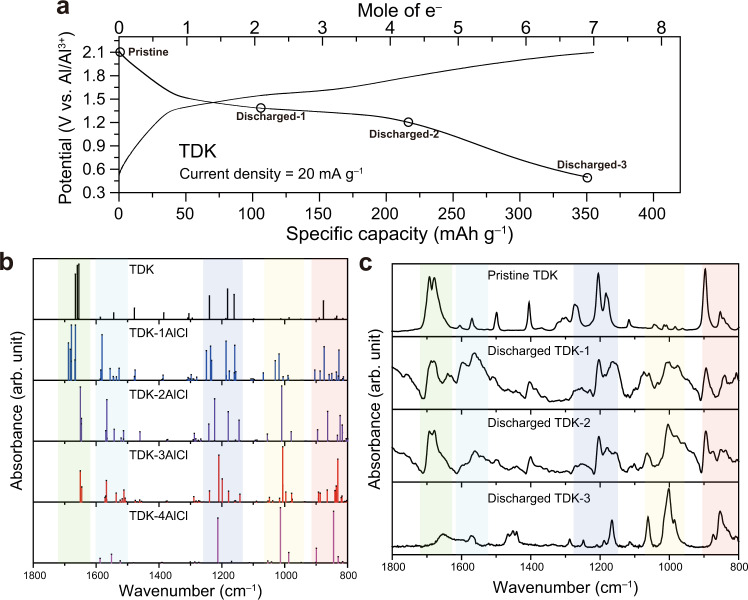
Ex situ FT-IR characterisation of TDK. **a** Galvanostatic voltage profiles of TDK at the current density of 20 mA g^−^^1^. **b** Calculated IR vibration peaks of TDK when bound with AlCl^2+^ ions. **c** Measured FT-IR absorbance spectra of the TDK electrode. The discharging points at which the measurements were carried out are indicated in (**a**).

Figure 3a shows the charge–discharge voltage profile of TDK when a current density of 20 mA g^−^^1^ was applied. TDK showed a plateau at approximately 1.3 V vs. Al/Al^3+^ which is similar to that of PQ (1.4 V vs. Al/Al^3+^). The reversible capacity of TDK at this current density was 350 mAh g^−1^, which corresponds to the storage of 7e^−^ per molecular unit. This capacity is far beyond 203 mAh g^−1^, which corresponds to the storage of 4e^−^ when all of the carbonyl groups of TDK are utilised to bind with monovalent ions. This strongly hints that the storage mechanism dominantly involves divalent ions. Remarkably, the specific capacity of TDK is also far above those of other materials reported as AIB cathodes to date, revealing the beneficial effect of divalent ion storage.

We monitored the change in functional groups during discharging by carrying out ex situ FT-IR spectroscopy and compared the results with the calculated IR vibration peaks. The FT-IR measurements of TDK were conducted after the second cycle at a current density of 20 mA g^−1^. Regarding the calculated peaks, we first confirmed their reliability by checking that the main peaks of TDK were in good agreement with the calculated ones (Supplementary Fig. 3a). Based on the confirmed reliability, we characterised the TDK electrodes at the different discharged states marked in Fig. 3a. These measurement points were set to enable the states of TDK to be captured after binding with every AlCl^2+^ ion. Based on this analysis, the following five features are noticeable from the viewpoint of both the simulation (Fig. 3b) and the experiments (Fig. 3c): (1) The peak intensity of the C=O asymmetric stretching modes (Supplementary Video 1) of TDK at 1686 cm^−1^ gradually decreased as a result of binding with an increasing number of AlCl^2+^ ions (green shades in Fig. 3b, c). (2) The peaks of the C–C and C=C stretching modes near the C=O that binds with the AlCl^2+^ ion (Supplementary Videos 2 and 3) were observed at 1581 and 1527 cm^−1^ for TDK-1AlCl, but gradually disappeared as more carbonyl groups formed bonds with AlCl^2+^ ions (cyan shades in Fig. 3b, c). (3) The peak of the C–C and C=C stretching modes (Supplementary Video 4) of TDK at 1180 cm^‒1^ was shifted to 1210 cm^−1^ in the case of TDK-4AlCl (Supplementary Video 5) (navy shades in Fig. 3b, c). (4) The peak at 1013 cm^−1^ corresponding to the C–C and C=C stretching modes and C=O stretching modes over the entire macrocycle appeared (Supplementary Video 6) and grew (Supplementary Video 7) to become the main peak of TDK-4AlCl (yellow shades in Fig. 3b, c). (5) The peak of the C–H wagging and C=O asymmetric stretching modes (Supplementary Video 8) of TDK at 880 cm^−1^ was replaced by the peak of TDK-4AlCl at 844 cm^−1^, corresponding to its C–H wagging and O–Al stretching modes (Supplementary Video 9) (red shades in Fig. 3b, c). For all of these observations, the calculations and actual analyses corresponded well indeed. The main peaks of discharged TDK were preserved after 5 cycles, indicating the robust nature of the reaction with AlCl^2+^ ions (Supplementary Fig. 5b). In addition, the calculated IR vibration peaks of TDK upon binding with AlCl_2_^+^ ions were conspicuously mismatched with the experimental ones (Supplementary Fig. 6), further supporting that the storage mechanism of TDK is based on AlCl^2+^ ions.

### Electrochemical performance of aluminium batteries

To minimise the dissolution of active molecules into the electrolyte, we infiltrated TDK into the pores of activated carbon (AC) by simply replacing carbon black with AC as the conductive agent. This choice of conductive agent was synergetic with the high solubility of TDK in *N*-methyl-2-pyrrolidone (NMP), a slurry solvent commonly used in battery fabrication, such that TDK naturally penetrated the pores of AC while the slurry was being mixed and dried. We first indirectly recognised the infusion of TDK into the pores of AC by the fact that the original yellowish colour of TDK powder was not observed after the evaporation of NMP (Supplementary Fig. 7). Scanning electron microscopy (SEM) provided more direct verification of the impregnation of TDK. The morphology of AC was restored in the case of the TDK-AC blend after the evaporation of NMP, whereas TDK was found to be recrystallised separately when blended with nonporous carbon materials such as Super P and multiwalled carbon nanotubes (MWCNTs) (Supplementary Fig. 8).

Further evidence that TDK had penetrated the pores of AC was obtained by recording the adsorption-desorption isotherms using N_2_ gas (Supplementary Fig. 9). Unlike the TDK-Super P and TDK-MWCNT blends, the specific surface area (SSA) of the TDK-AC blend (705 m^2^ g^−1^) was significantly smaller than that of pristine AC (2347 m^2^ g^−1^) as a result of the pores being filled with TDK. The infiltration of TDK was additionally detected by X-ray diffraction (XRD) analysis (Supplementary Fig. 10). Although the characteristic XRD peaks were observed for the TDK-AC blend, their intensities were weakened owing to the confining effect in the pores. When a TDK solution is condensed by allowing the solvent to evaporate, TDK alone tends to crystallise as indicated by its XRD peaks (Supplementary Fig. 10). However, when confined within a space with comparable dimensions to that of TDK clusters, molecule-to-wall interactions could perturb intermolecular interaction and therefore the crystallinity of TDK. The confinement effect was much less significant with the TDK-Super P and TDK-MWCNT blends because these carbon materials do not form pores with such small dimensions. Note that low crystallinity is usually beneficial as it enables active molecules to sustain themselves against dissolution in the electrolyte during cycling; by contrast, molecules with high crystallinity are more vulnerable to solvation by the electrolyte solvent^38,39^.

**Fig. 4:**
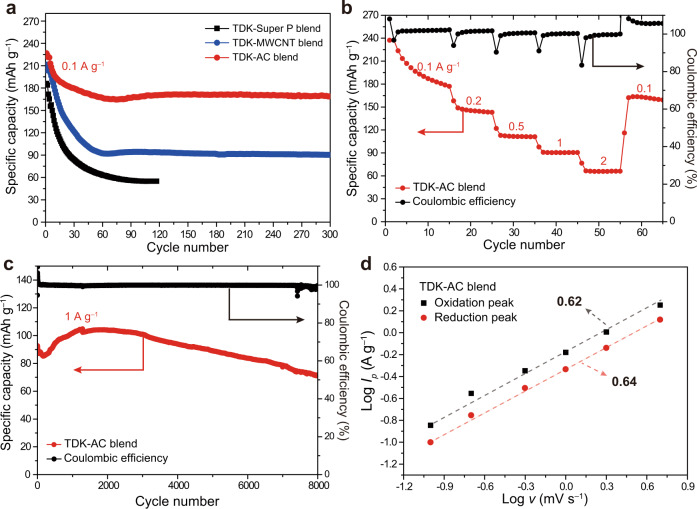
Electrochemical performance of TDK. **a** Cycling performance of TDK with different carbon materials. **b** Rate capability of TDK-AC blend. **c** Long-term cycling performance of TDK-AC blend at a current density of 1 A g^−1^. **d** Log *v*-log *I*_*p*_ plots to extract the *b*-values based on the relation *I*_*p*_ = *av*^*b*^.

The electrochemical performance of TDK was evaluated by various electrochemical analyses (Fig. 4). It is noteworthy that AC alone exhibited a low specific capacity of 40 mAh g^−1^, implying that its contribution to the capacity of TDK-AC blend is not substantial (Supplementary Fig. 11). To this end, TDK-Super P, MWCNT, or AC blends were subjected to galvanostatic cycling tests (Fig. 4a). All of the TDK-carbon blends showed similar specific capacities in their first two cycles (Supplementary Fig. 12). The application of a current density of 0.1 A g^−1^ resulted in the rapid decay of the capacities of the TDK-Super P and MWCNT blends to 55 and 90 mAh g^−^^1^ after the first 50 cycles, respectively, due to the severe dissolution problem. In the case of the TDK-AC blend, although it showed a capacity decrease from 226 to 170 mAh g^−^^1^ during the first 50 cycles, which probably originated from the dissolution of a small portion of recrystallised TDK on the surface of AC as detected in the XRD results (Supplementary Fig. 10), it retained a remarkably high capacity of 170 mAh g^−1^ after 300 cycles, revealing the confined TDK to be highly stable. To note, the capacity decay at the very low current density was mainly ascribed to irreversible oxidation of the electrolyte at high voltages in addition to the inevitable dissolution of discharged TDK (Supplementary Fig. 13). The superior performance of the TDK-AC blend was also observed in rate capability tests (Fig. 4b). An increase in the current density from 0.1 to 0.2, 0.5, 1, and 2A g^−1^ maintained the capacity from 185 to 145, 111, 90 and 66 mAh g^−1^, respectively. When the current density was returned to 0.1 A g^−1^, the capacity of 165 mAh g^−1^ was recovered, showing the sustainable nature of the TDK-AC blend against a sweep across various current densities. The cycling performance of the TDK-AC blend was also stable such that when cycled at 1 A g^−^^1^, the cell preserved 78.0% of its original capacity even after 8000 cycles (Fig. 4c and Supplementary Fig. 14). The initial capacity increase at the high current density was attributed to an activation process required for electrolyte infiltration, which is supported by the charge transfer resistance (*R*_ct_) that decreased during initial cycles (Supplementary Fig. 15).

The charge storage mechanism of electrode materials can be elucidated by scan rate-dependent cyclic voltammetry (CV) measurement, in which the capacity contribution is divided into faradaic and non-faradaic processes. The faradic process relates to a (de)intercalation and charge-transfer process, and the non-faradaic process relates to capacitive ion adsorption in the electric double layer^40,41^. The contributions from these two processes can be identified by analysing the CV profiles at various scan rates according to the following relation between the peak current (*I*) and the scan rate (*v*)

$$I=a{v}^{b}$$

in which the value of *b* can be determined by the slope of the (log *v*)–(log *I*) plot. Recall that *b* = 0.5 corresponds to a diffusion-controlled process, whereas *b* = 1 exclusively indicates a capacitive-limited process. Using the redox peaks in the CV profiles (Supplementary Fig. 16a), we plotted the (log *v*)–(log *I*_*p*_) of the TDK-AC electrode (Fig. 4d). The *b*-values of the reduction and oxidation were 0.64 and 0.62, respectively, implying that a large portion of charge storage is diffusion-controlled and that the diffusion kinetics of AlCl^2+^ ions in TDK may not be very efficient.

### Effect of the valence state of the carrier ion on the electrochemical performance

The extent to which the kinetics depended on the charge carrier ions was determined by preparing graphite and PQ electrodes, which are known^19,37^ to exploit AlCl_4_^−^ and AlCl_2_^+^ ions for charge storage, respectively. The *b*-values of graphite and PQ were extracted from the redox peaks in the CV profiles (Supplementary Fig. 16b, c). The *b*-values related to the reduction and oxidation of graphite were 0.92 and 0.91, respectively, whereas those of PQ were 0.72 and 0.87, respectively (Supplementary Fig. 17). Although both graphite and PQ utilise monovalent ions as carrier ions, the higher *b*-values and diffusion kinetics of graphite are attributed to its intrinsically high electrical conductivity. The fact that both graphite and PQ have *b*-values higher than those of TDK supports our findings that TDK stores divalent ions (AlCl^2+^) and explains its relatively sluggish diffusion.

**Fig. 5:**
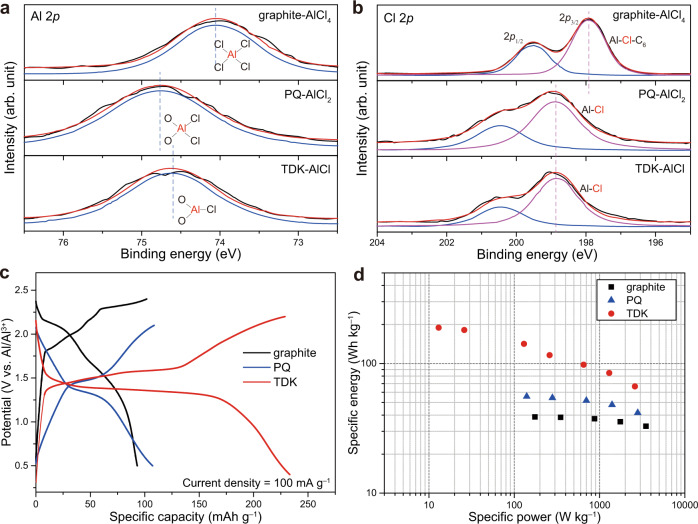
Comparison of cathode materials exploiting AlCl_4_^−^, AlCl_2_^+^ and AlCl^2+^ ions. **a**, **b** XPS curves of graphite-AlCl_4_, PQ-AlCl_2_ and TDK-AlCl; **a** Al 2*p* and **b** Cl 2*p* branches. **c** Galvanostatic voltage profiles of graphite, PQ and TDK when the current density is 100 mA g^−1^. **d** Ragone plots of cells based on graphite, PQ and TDK. The required amount of electrolyte was taken into account for this calculation.

The effect of the functional groups on the charge carrier ions was examined by X-ray photoelectron spectroscopy (XPS) to analyse the graphite, PQ, and TDK electrodes. Because the binding energy of the core electrons is sensitive to the chemical environment of the corresponding element, the XPS peak shift allows us to clarify the binding structures of the charge carrier ions. To note, all the XPS peaks were referenced with respect to the C‒C bond at 284.8 eV in the C 1*s* branch (Supplementary Fig. 18d). Prior to analysing the binding structures, we first verified that the charge carrier ions stored in the graphite, PQ, and TDK were AlCl_4_^−^, AlCl_2_^+^ and AlCl^2+^ based on the fact that the atomic ratios of Cl to Al were 3.93, 1.75 and 0.97, respectively (Supplementary Fig. 18). The atomic ratio in TDK-AlCl was further confirmed by energy dispersive spectroscopy mapping analysis (Supplementary Fig. 19). In the Al 2*p* branches (Fig. 5a), the peaks at 74.1, 74.8 and 74.6 eV were assigned to graphite-AlCl_4_, PQ-AlCl_2_ and TDK-AlCl, respectively. It is instructive to note that the binding energy shifts to a higher value when the element is bonded with functional groups or elements with higher electronegativity. On the basis of this rationale, the location of the Al peak of PQ-AlCl_2_ at higher binding energy compared to that of graphite-AlCl_4_ can be understood by taking into account that Al is bonded with the electronegative carbonyl groups of PQ. Based on the same logic, the slightly lower binding energy of the Al peak of TDK-AlCl compared with that of PQ-AlCl_2_ can be explained by the fact that the Al in TDK-AlCl is bonded with a single Cl atom. On the other hand, in the Cl 2*p* branches (Fig. 5b), the peaks at 198.9 eV were shared by PQ-AlCl_2_ and TDK-AlCl, as the Cl atoms in PQ-AlCl_2_ and TDK-AlCl are both bound solely to the Al atom. In the case of graphite-AlCl_4_, however, the peak of Cl shifted downward to 197.9 eV because AlCl_4_ interacts with the graphitic layers to a certain degree.

The energy density of electrode materials is by and large determined by their operating voltage and specific capacities. Even though this metric is generally valid, the energy density of an AIB should be carefully evaluated. In an AIB cell, the charge carrier ions used in the anode and cathode are different, which implies that at least one carrier ion must be supplied by the electrolyte and the amount of electrolyte should certainly be taken into consideration in the energy density calculation. Figure 5c shows the charge–discharge voltage profiles of the graphite, PQ and TDK cells. When considering only the operating voltage and specific capacity, the graphite, PQ and TDK cells deliver energy densities of 170, 168 and 278 Wh kg^−^^1^, respectively. However, when the amounts of electrolyte are taken into account, the energy densities are changed as marked in Fig. 5d. In this sense, the charge carrier ion plays a role as the Al-to-Cl ratio determines the amount of the electrolyte. Details of the calculation are given in the Supplementary Note. Although the rate performance of the graphite and PQ cells was higher as they take advantage of the more facile diffusion of the carrier ions (Supplementary Fig. 20), their energy densities are only 38 and 55 Wh kg^−1^, respectively. On the contrary, the TDK cell exhibited far higher energy density of 189 Wh kg^−1^ and power density of 2600 W kg^−1^ by utilising the divalent AlCl^2+^ ions and this performance is linked to the aforementioned superior specific capacity of the TDK electrode. Although the specific energy values would need to be further improved to attain practical competitiveness, the present study demonstrates the impact of exploiting multivalent carrier ions and the relevance of active material design.

## Discussion

The core advantage of AIBs is its capability to store multivalent carrier ions to increase the specific capacity of electrode materials. Unfortunately, nearly all the cathode materials reported to date operate on the basis of the storage of monovalent complex ions such that the true benefit of AIBs has not been fully taken advantage of. We introduced the ‘relative stability’ of discharged states bearing carrier ions with different valence states as a key parameter to determine the principal carrier ion for active diketone molecules in AIBs. The structural stability of the discharged state with monovalent ions is particularly determined by the resonance effect of their benzene rings to stabilise the radical state. As predicted by the low ring-to-carbonyl group ratio, the TDK radical, upon binding with the monovalent ions, experiences moderate resonance stabilisation, rendering divalent ion storage dominant. Based on this design rationale toward achieving divalent ion storage, TDK delivers an extraordinarily high specific capacity of 350 mAh g^−1^ as an AIB cathode material with an operating voltage of 1.3 V (Al/Al^3+^) as well as a lifetime that exceeds 8000 cycles. In a greater context, our study suggests ways in which to design diketone molecules to activate the multivalent ion storage mechanism; that is, to destabilise the radical formed upon reaction with monovalent ions.

## Methods

### Material preparation

Terephthalaldehyde (20.1 g, 150 mmol), water (450 mL) and 2-methoxyethanol (450 mL) were added to a 1 L round-bottom flask equipped with a reflux condenser. The resulting stirred suspension was purged with nitrogen and heated to reflux under nitrogen to dissolve the starting material. NaCN (735 mg, 15.0 mmol) was then added and the reaction mixture was maintained under reflux for 3 days. The precipitated cyclotetrabenzoin intermediate was isolated by filtration while the reaction was still hot (using a preheated sintered glass funnel) and was then washed several times with water, methanol, and diethyl ether. After drying in vacuo, 10.1 g (18.8 mmol) of the crude intermediate was obtained, and this was placed in a 1 L round-bottom flask and suspended in concentrated HNO_3_ (70%, 380 mL). The flask was equipped with a reflux condenser and the reaction was slowly heated to reflux, resulting in the initial intense formation of nitrous fumes. After 60 h at reflux, the reaction was cooled to room temperature and water (380 mL) was added carefully. The solid was isolated by filtration, washed twice with water, ethanol, and diethyl ether, and extracted with chloroform in a Soxhlet extractor for 3 days to dissolve the TDK and separate it from insoluble side products. Evaporation of the chloroform yielded pure TDK (5.24 g, 9.92 mmol) as a bright yellow powder in an overall yield of 26%. ^1^H NMR (400 MHz, DMSO-d_6_): *δ* = 7.90 (s, 16H) ppm; in accordance with the literature^36^. ^13^C NMR (101 MHz, DMSO-*d*_6_): *δ* = 194.5, 135.8, 130.5 ppm.

### Cell preparation and electrochemical measurements

The electrodes were prepared using the following procedures. TDK, the conductive agent, and poly(vinylidene difluoride) (PVDF, Arkema) binder were dispersed in *N*-methyl 2-pyrrolidone (NMP) in a weight ratio of 50:40:10. The slurry was cast onto tantalum foil (99.95%, Thermo Fisher) by using the doctor blade technique, and the cast electrodes were dried under vacuum at 80 °C for 24 h. The conductive agents were Super P, MWCNTs or AC (AC0830, Asahi Organic Chemicals). The mass loading of TDK in each electrode was 1.5 mg cm^−2^. For the PQ electrodes, PQ, AC and PVDF binder were employed in a weight ratio of 50:40:10. For the graphite electrodes, graphite (N006, Digichem), Super P and PVDF binder were used in a weight ratio of 80:10:10. The aluminium electrolyte was prepared by slowly adding aluminium trichloride (AlCl_3_) to ethyl-3-methylimidazolium chloride (EMImCl) in a molar ratio of 1.5:1 (AlCl_3_:EMImCl). The electrochemical measurements were conducted by using modified Swagelok-type cells in which a stainless-steel rod and a glassy carbon rod were used as current collectors for the anode and cathode, respectively. The cells were composed of an aluminium metal foil anode, a glass fibre membrane (GF/D Whatman) and a cathode. The entire cell was assembled inside an argon-filled glove box. Galvanostatic measurements and cyclic voltammetry were performed using a battery cycler (WBCS3000L, Wonatech) at 25 °C. The Coulombic efficiency was defined as the ratio of charge capacity to discharge capacity because each cell began to operate by discharging.

### Characterisation

TDK was analysed by NMR spectroscopy and TGA to verify the functional groups and purity. Ex situ characterisations were conducted by analysing the electrode samples by FT-IR (JASCO FT/IR-6700). The morphologies of the TDK electrodes comprising various carbon materials were characterised by using field-emission scanning electron microscopy (JSM-7800F Prime, JEOL) on the instrument housed at the National Center for Inter-University Research Facilities (NCIRF) at Seoul National University. The SSAs of TDK electrodes with various carbon materials were obtained from N_2_ adsorption-desorption measurements by using a porosity analyser (Micromeritics, 3FLEX) operating at 87 K. The XRD profiles were obtained by using an X-ray diffractometer (SmartLab, Rigaku) based on Cu-Kα (λ = 0.15406 nm) radiation. The chemical binding states of discharged graphite, PQ and TDK were characterised by XPS (Sigma Probe, Thermo VG Scientific) with an Mg Kα line as the X-ray source. All the electrode samples for ex situ characterisations were washed with 1,2-dichloroethane in an argon-filled glove box, followed by drying under vacuum for 3 h.

### DFT calculations

Geometrical optimisations, energy calculations and atomic charge analysis were carried out without symmetry restriction using the B3LYP hybrid density functional implemented in the GAUSSIAN 09 software package^42^. The 6–31 + *G*(*d*) basis sets were adopted for all the atoms, and the polarisation continuum model with the ethanol parameter and dielectric constant (*ε* = 24.6) was used to implicitly include solvent effects in the calculations. Formation energies were obtained by determining the energy difference between the sum of the energies of individual species and the energies of coordinated species. Natural bond orbital analysis was employed to evaluate the charge variation. IR vibration modes were obtained from frequency calculations, and the frequencies were scaled by 0.98 to match the experimental data.

## Supplementary information

Supplementary Information

Description of Additional Supplementary Files

Supplementary Video 1

Supplementary Video 2

Supplementary Video 3

Supplementary Video 4

Supplementary Video 5

Supplementary Video 6

Supplementary Video 7

Supplementary Video 8

Supplementary Video 9

## Data Availability

The data that support the findings of this study are available from the corresponding author upon reasonable request.
