# Supplementary material for: Tetradiketone macrocycle for divalent aluminium ion batteries
Source: Nat Commun. 2021 Apr 22;12:2386. doi: 10.1038/s41467-021-22633-y (PMC8062564; doi:10.1038/s41467-021-22633-y)
Supplement: Supplementary file 1 — Supplementary Information [file 41467_2021_22633_MOESM1_ESM.pdf]

Tetradiketone macrocycle for divalent aluminium ion batteries

Dong-Joo Yoo[1], Martin Heeney[2], Florian Glöcklhofer[2]* and Jang Wook Choi[1,3]*

[1]School of Chemical and Biological Engineering and Institute of Chemical Processes, Seoul National University, 1 Gwanak-ro, Gwanak-gu, Seoul 08826, Republic of Korea

[2]Department of Chemistry and Centre for Processable Electronics, Imperial College London, London W12 0BZ, UK

[3]Department of Materials Science and Engineering, Seoul National University, 1 Gwanak-ro, Gwanak-gu, Seoul 08826, Republic of Korea

*Corresponding author. E-mail: *f.glocklhofer@imperial.ac.uk, jangwookchoi@snu.ac.kr*

Supplementary Information

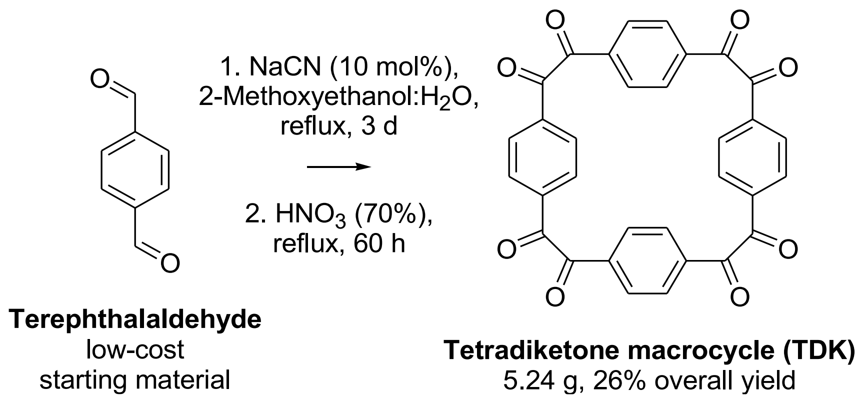

Supplementary Fig. 1 | Synthesis of TDK from low-cost terephthalaldehyde.

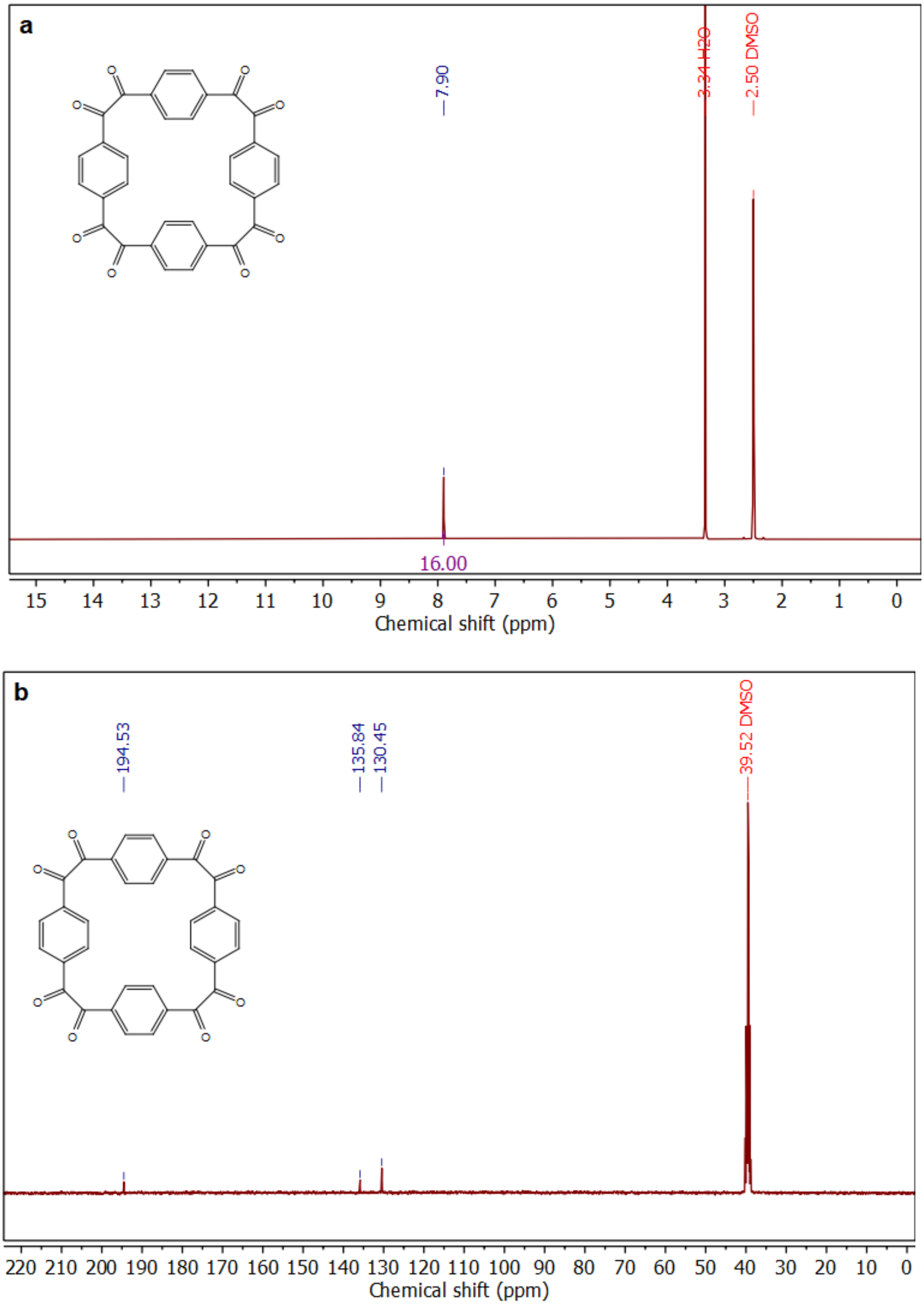

Supplementary Fig. 2 | a, ^{1}H NMR spectrum (400 MHz) of TDK in DMSO-d_6. **b**, ^{13}C NMR spectrum (101 MHz) of TDK in DMSO-d_6.

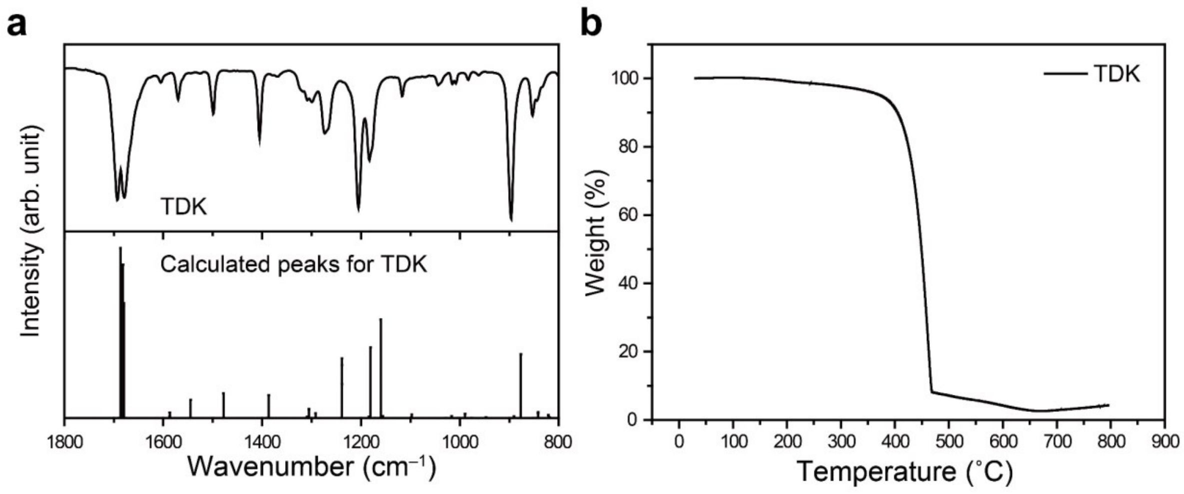

Supplementary Fig. 3 | **a**, FT-IR spectrum of TDK and its calculated peaks. **b**, TGA profile of TDK.

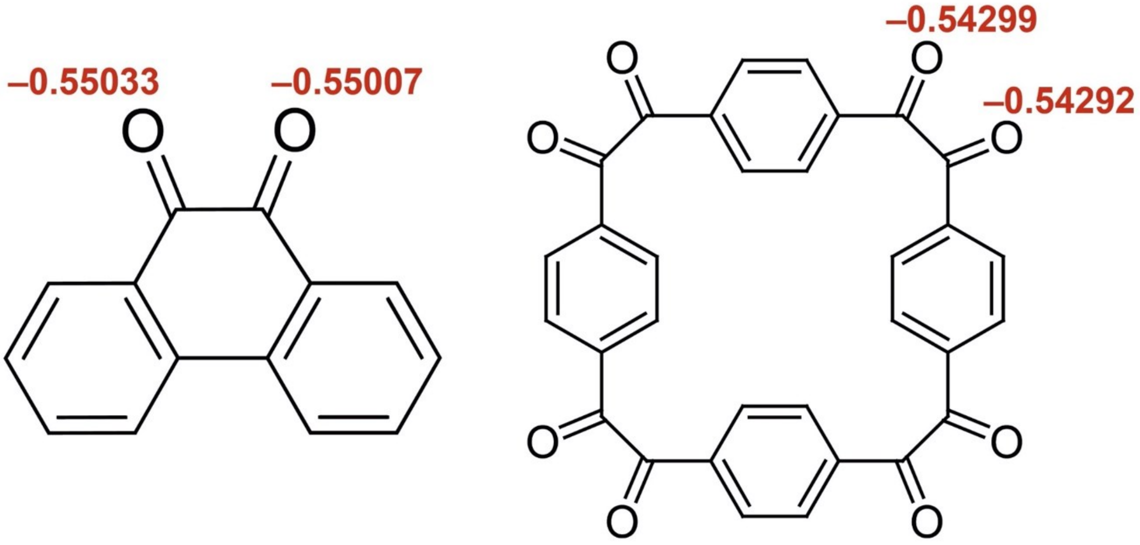

Supplementary Fig. 4 | Atomic charge analysis of pristine PQ and TDK.

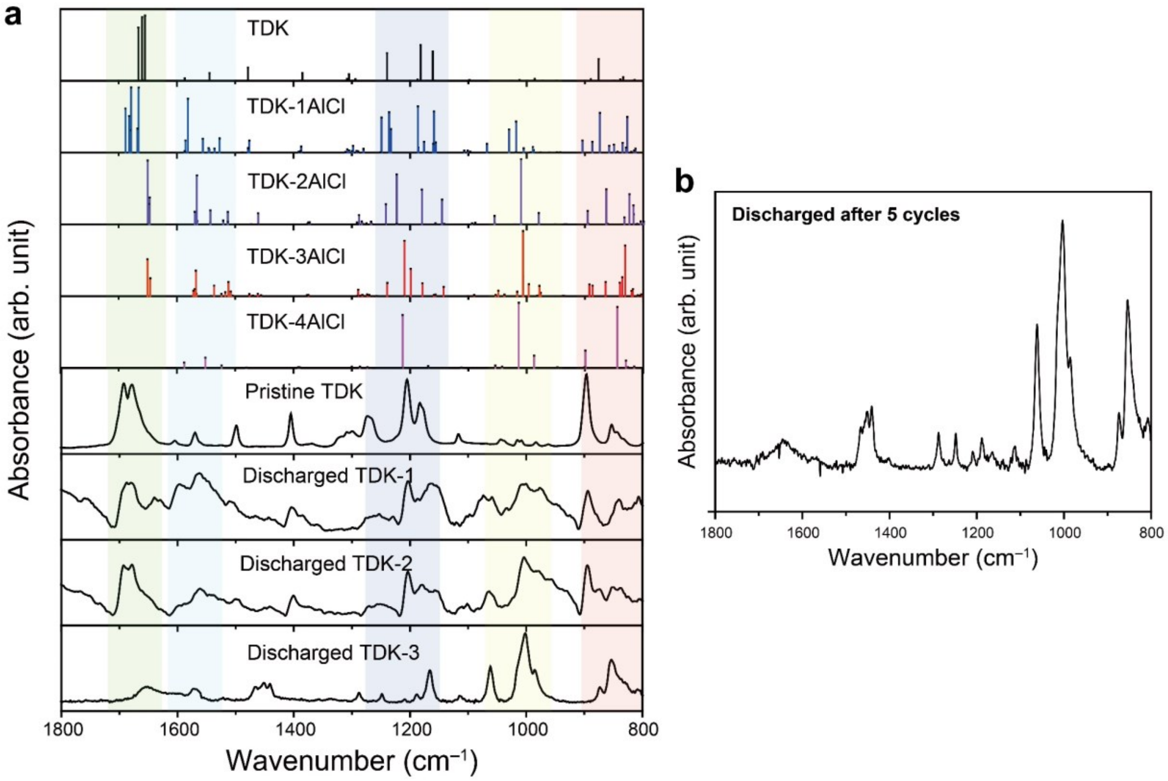

Supplementary Fig. 5 | (a) Direct comparison of calculated IR vibration peaks and measured FT-IR absorbance spectra of TDK electrode. (**b**) FT-IR absorbance spectra of discharged TDK electrode after 5 cycles at a current density of 0.2 A g^{-1}.

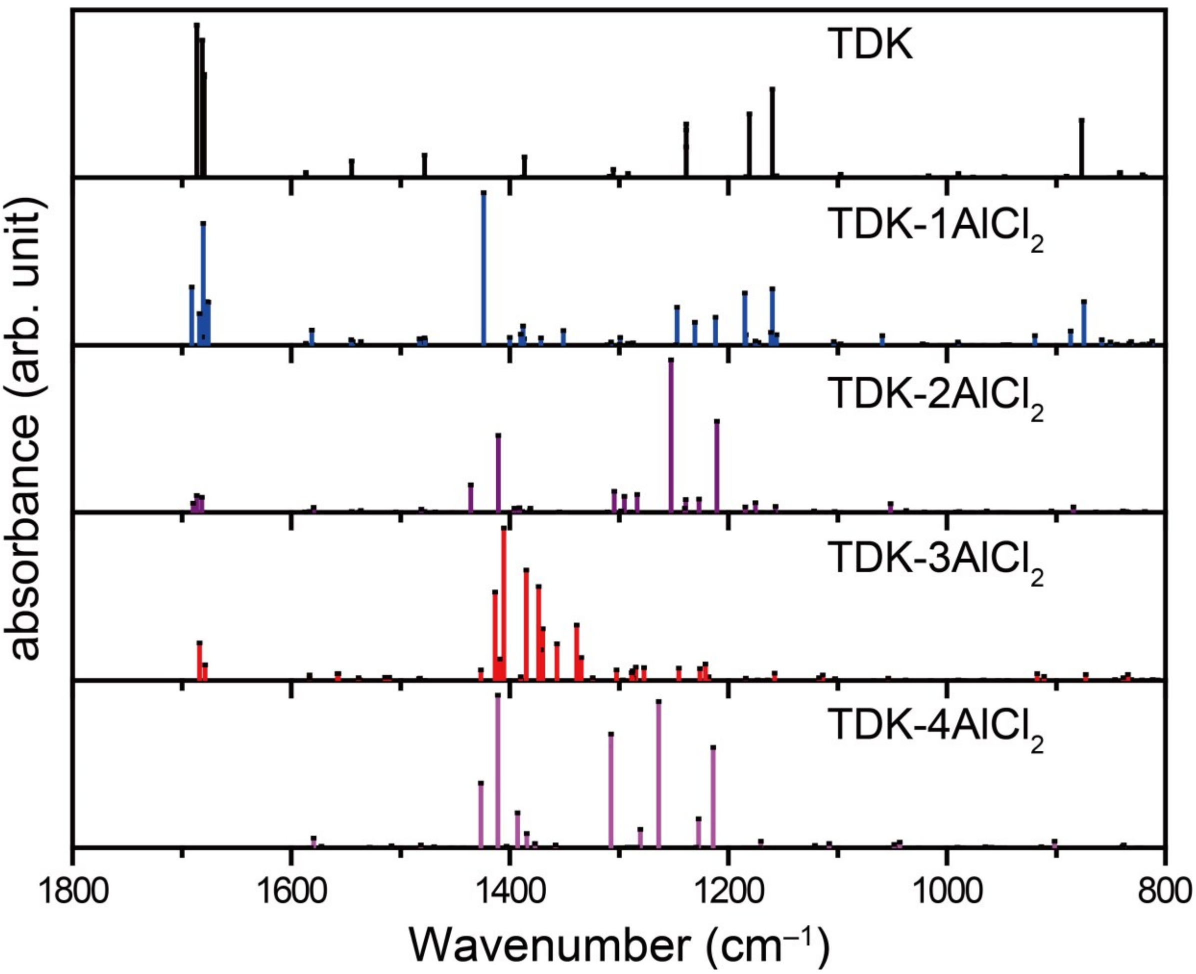

Supplementary Fig. 6 | Calculated IR peaks of TDK when bound with $AlCl_2^+$ ions.

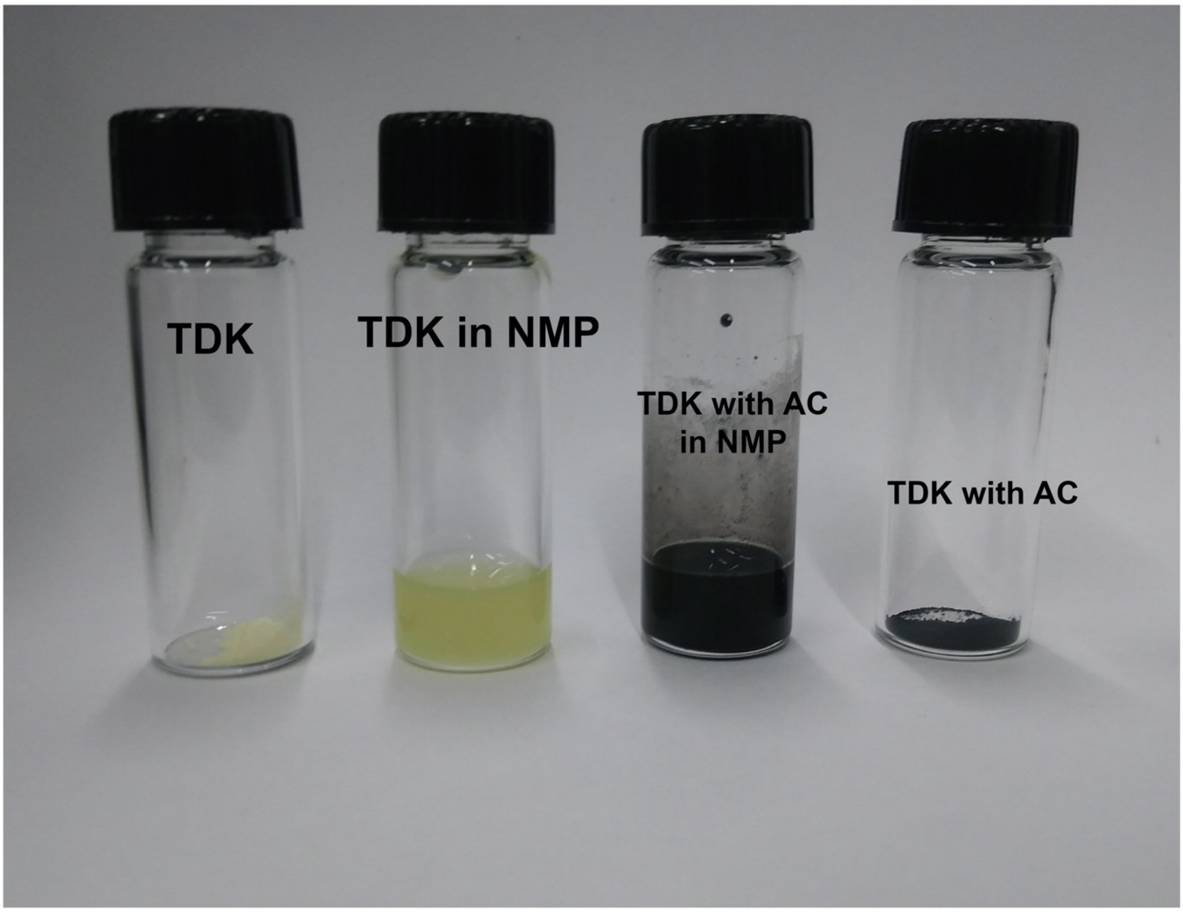

Supplementary Fig. 7 | Photograph of TDK at different stages of electrode preparation.

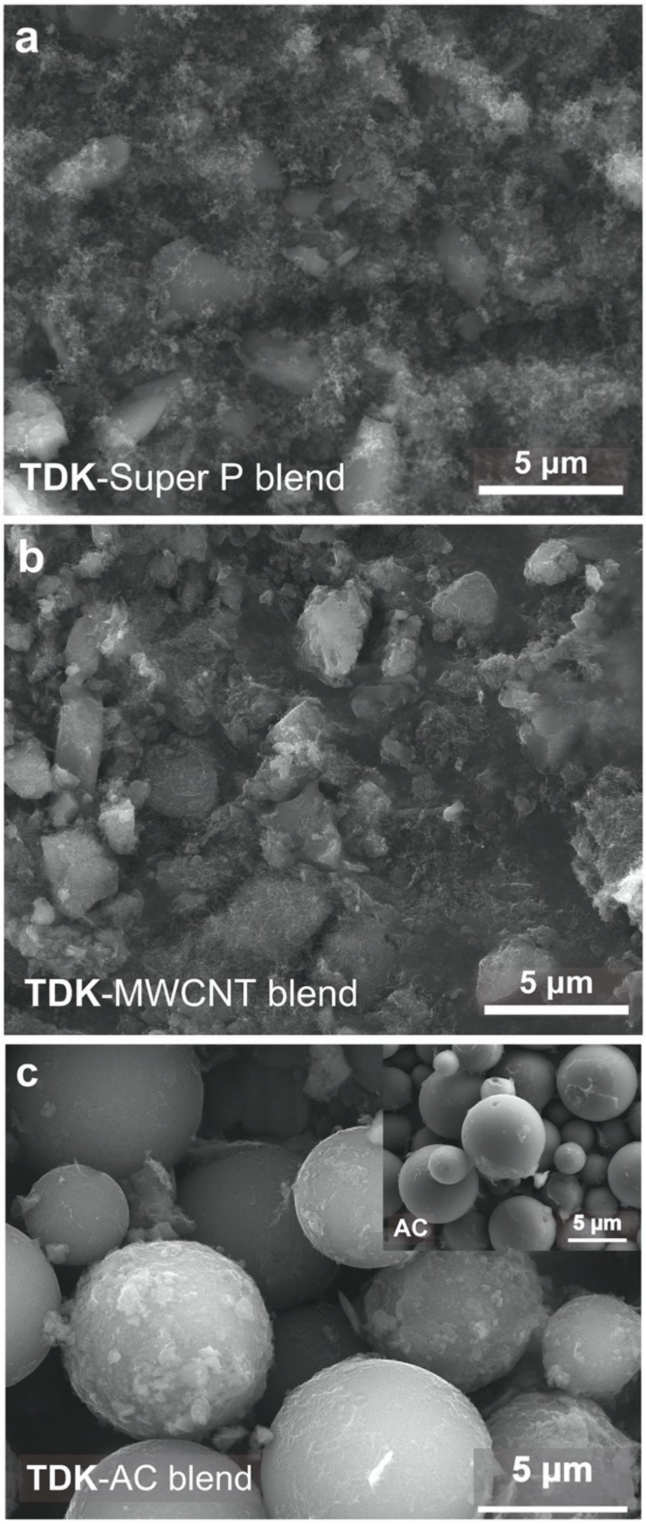

Supplementary Fig. 8 | SEM images of TDK with (**a**) Super-P, (**b**) MWCNT, and (**c**) AC.

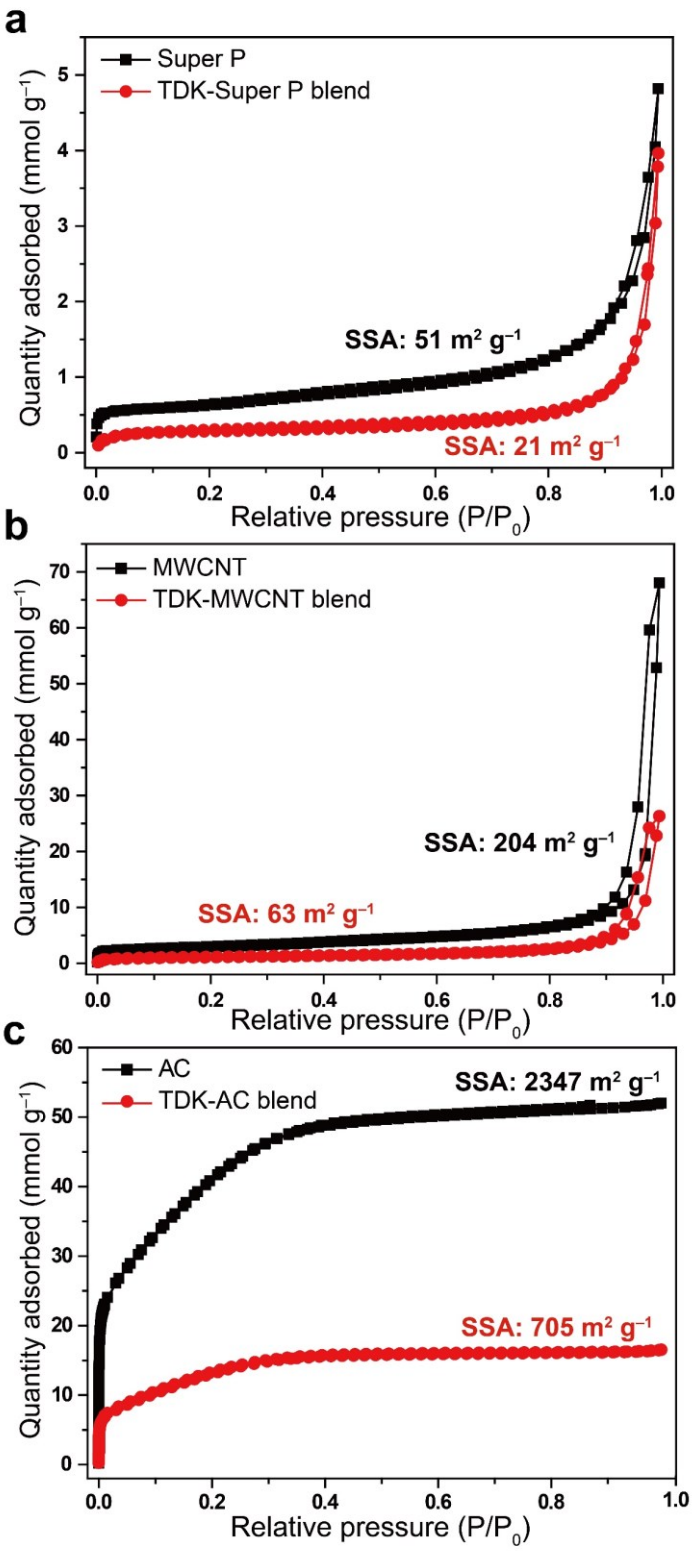

Supplementary Fig. 9 | Adsorption-desorption isotherms of TDK with (**a**) Super P, (**b**) MWCNT, and (**c**) AC.

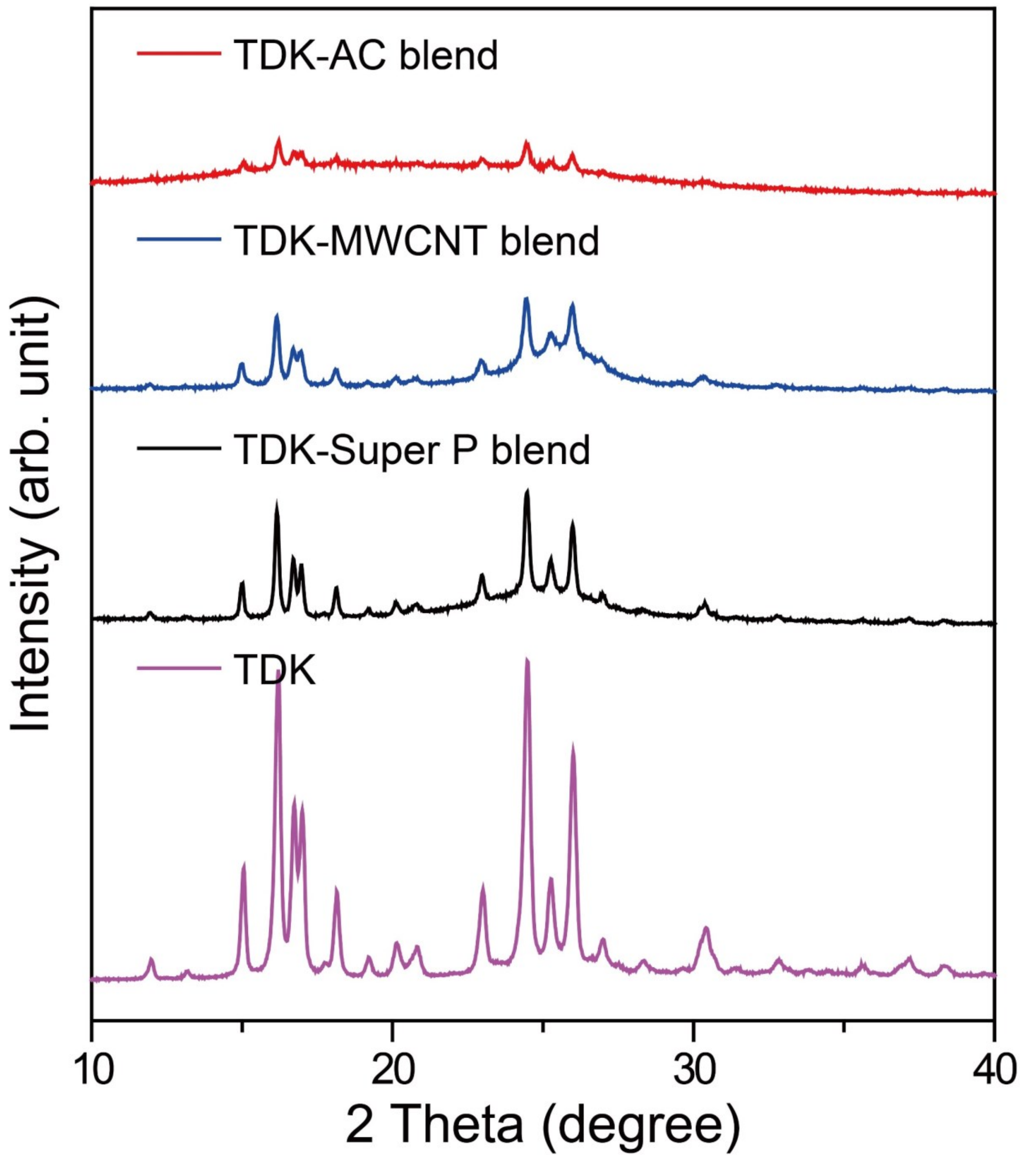

Supplementary Fig. 10 | XRD patterns of pristine TDK, and TDK-Super P, MWCNT, and AC blends.

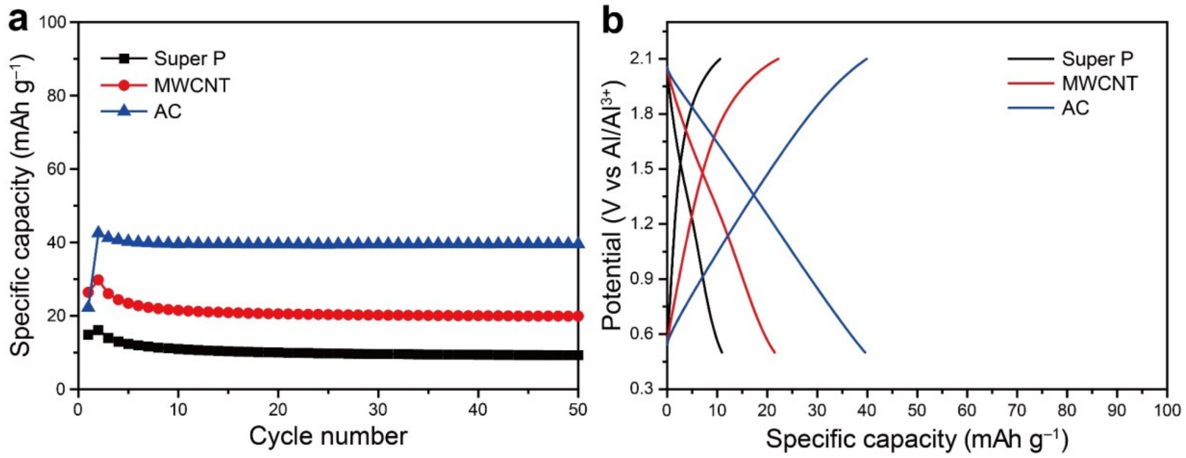

Supplementary Fig. 11 | (**a**) Cycling performance of different carbon materials at a current density of 0.1 A g^{-1} and (**b**) their voltage profiles at the 3rd cycle.

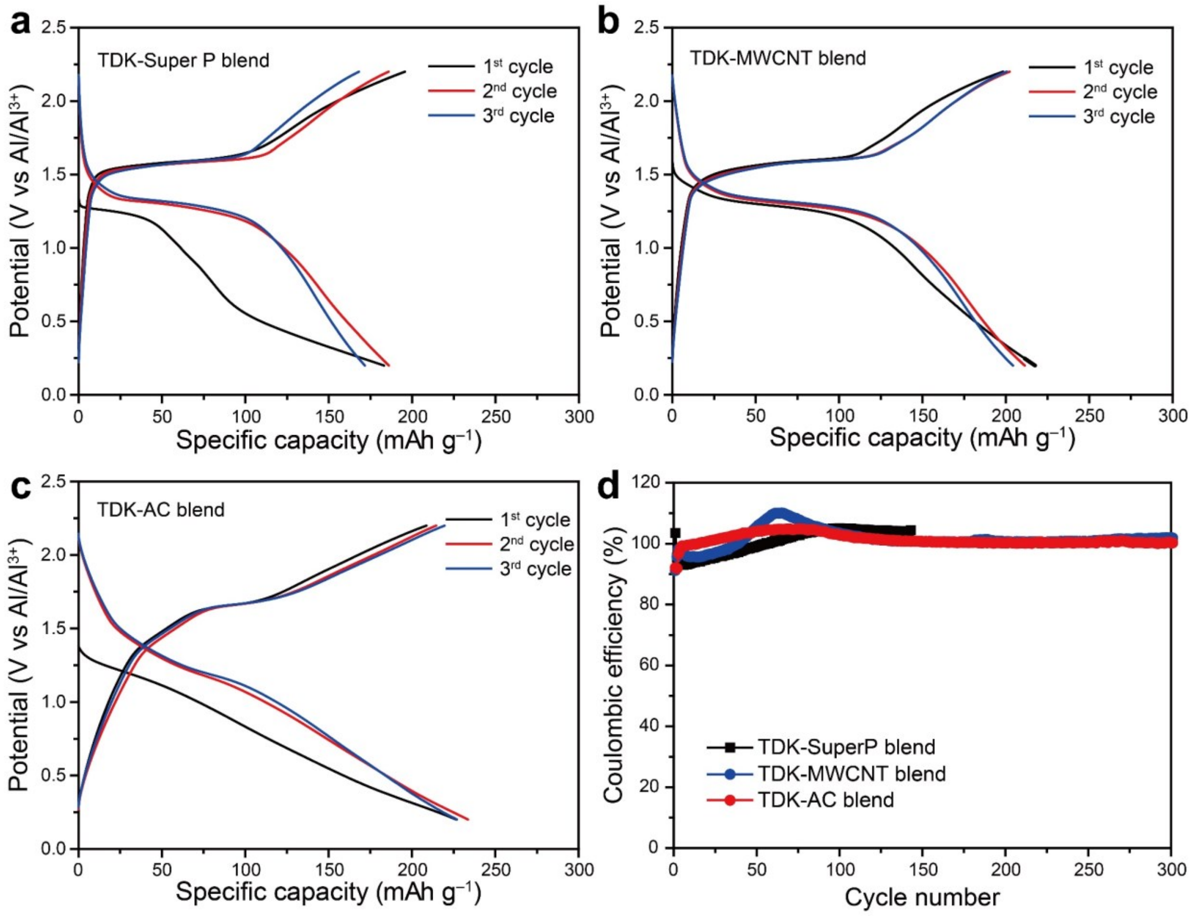

Supplementary Fig. 12 | Voltage profiles of the first three cycles of (**a**) TDK-Super P, (**b**) TDK-MWCNT, and (**c**) TDK-AC blends, and (**d**) their Coulombic efficiencies in Fig. 4a.

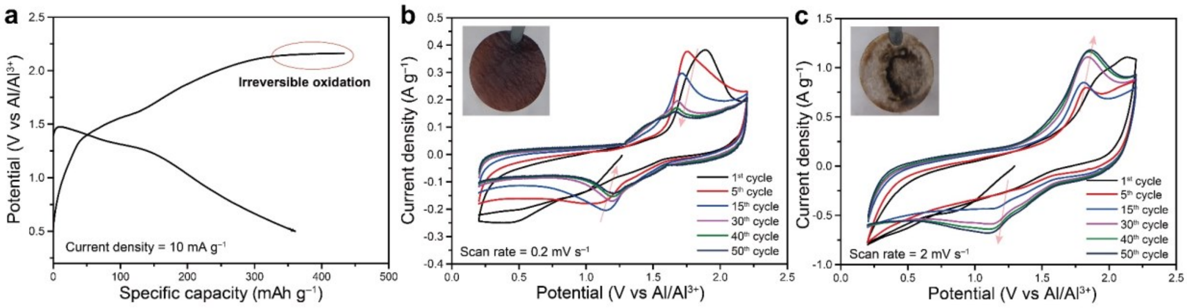

Supplementary Fig. 13 | Voltage profile of (**a**) TDK-AC blend at a current density of 10 mA g^{-1}. CV profiles of TDK-AC blend at a scan rate of (**b**) 0.2 and (**c**) 2 mV s^{-1}. Inset: digital photographs of the separators after CV cycling.

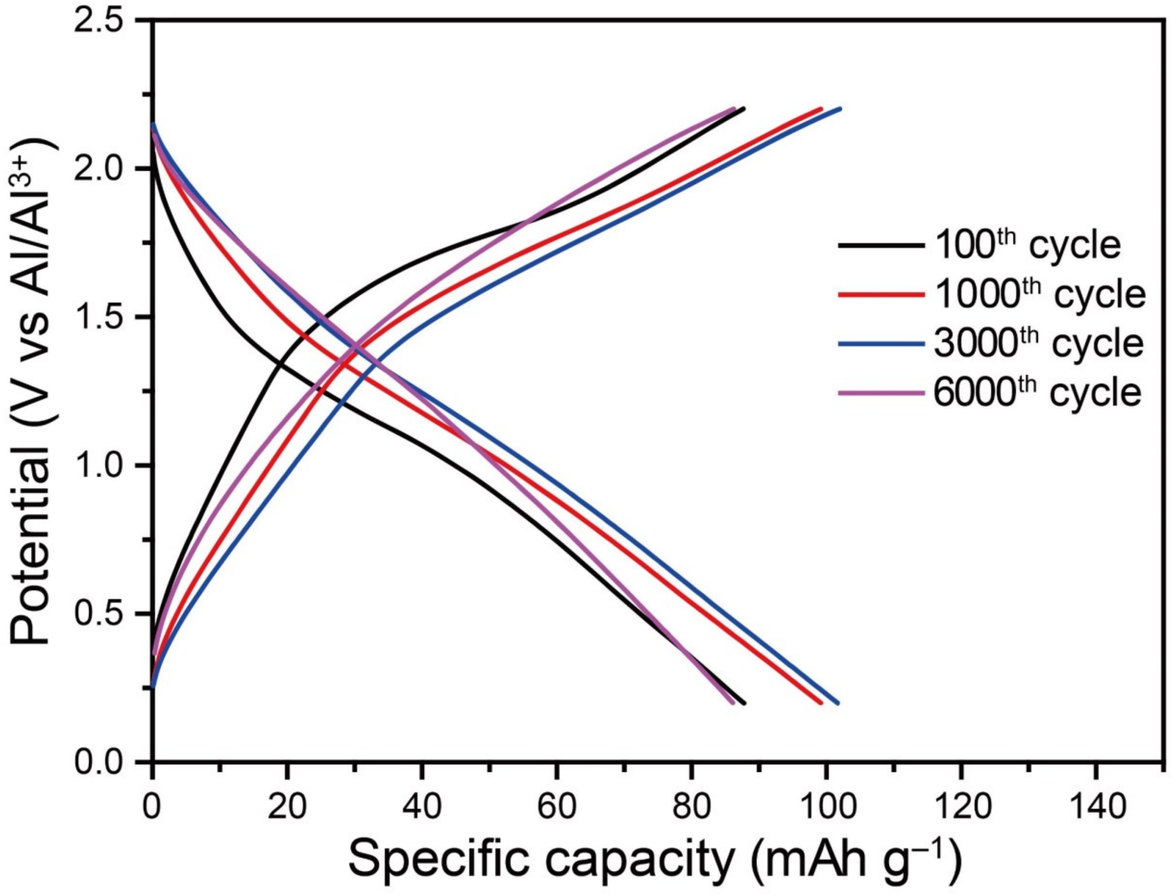

Supplementary Fig. 14 | Voltage profiles of selected cycles of TDK-AC blend in Fig. 4c.

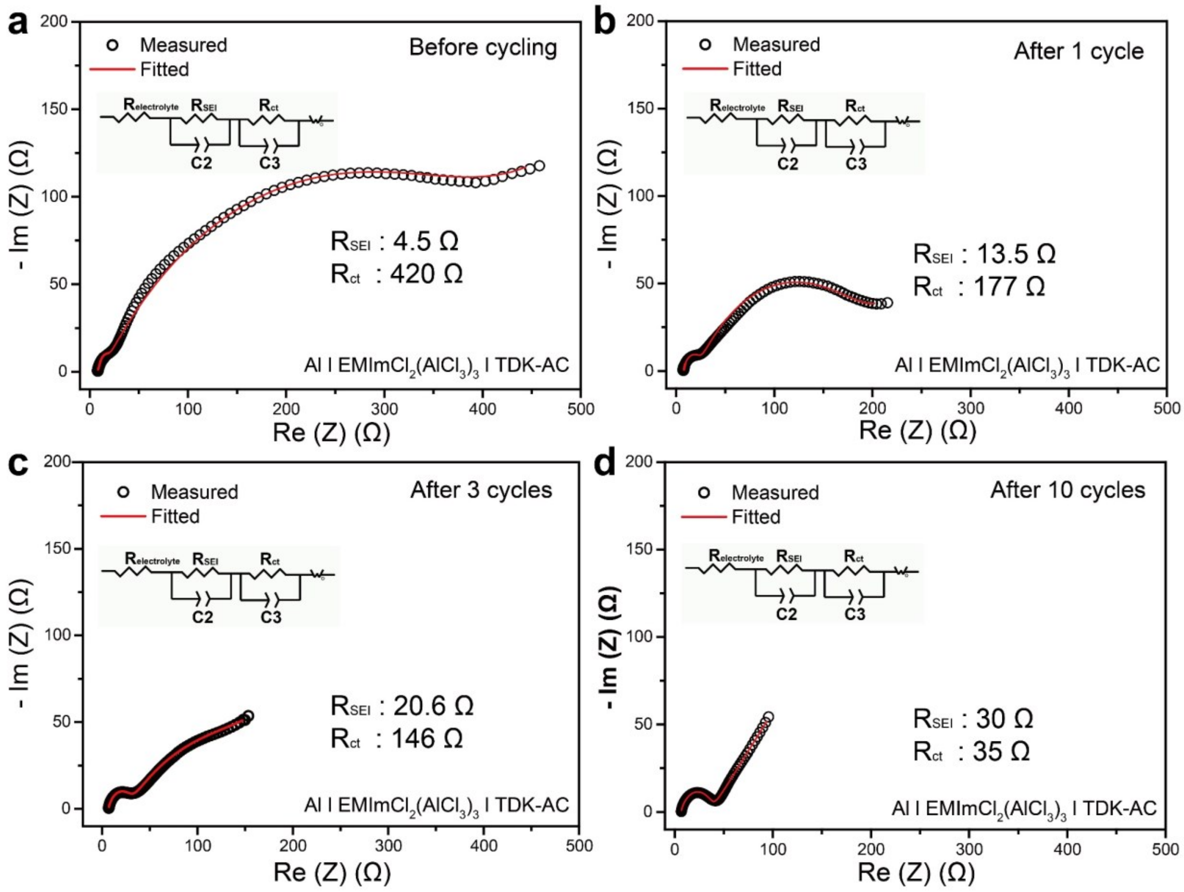

Supplementary Fig. 15 | Nyquist plots of TDK-AC blend (**a**) before cycling and after (**b**) 1, (**c**) 3, and (**d**) 10 cycles at a current density of 0.2 A g^{-1}.

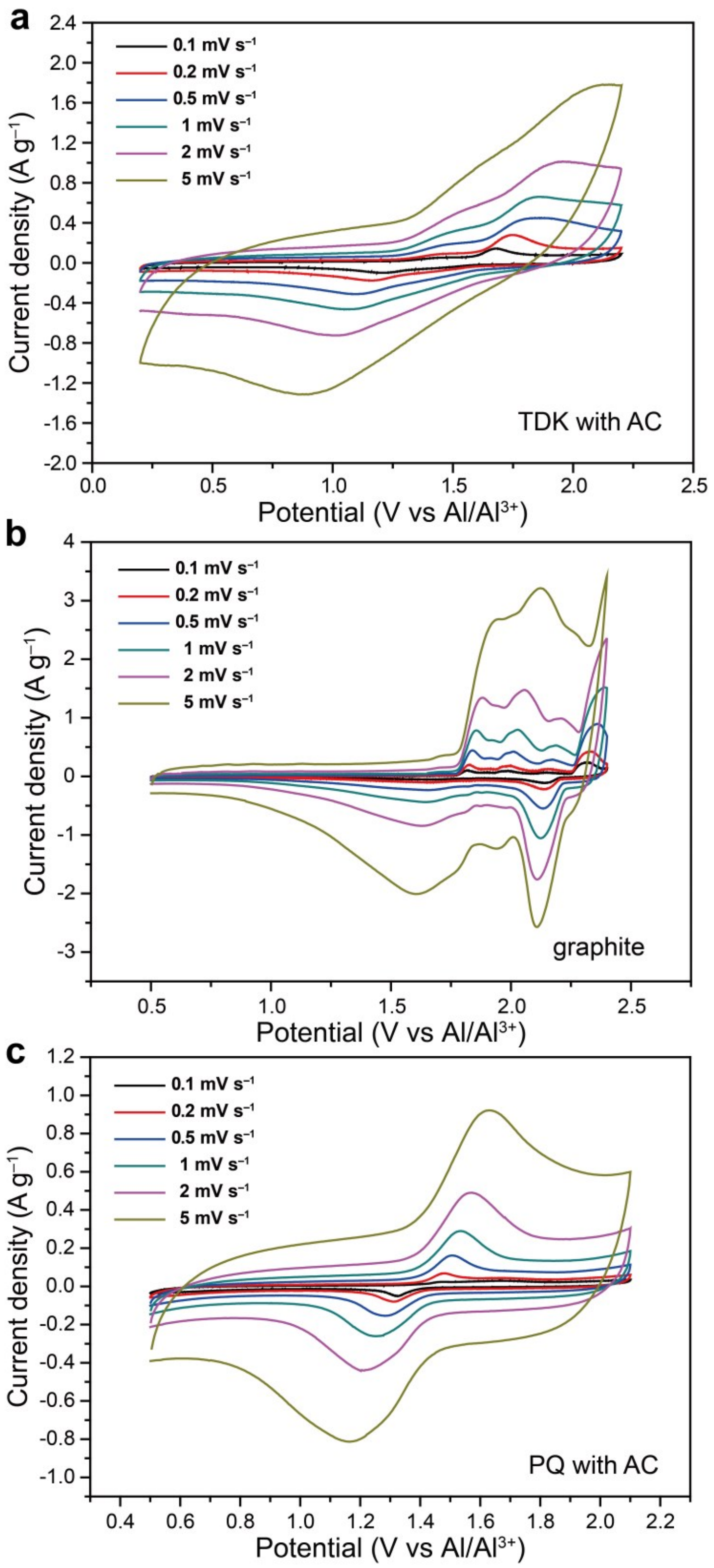

Supplementary Fig. 16 | CV profiles of (**a**) TDK, (**b**) graphite, and (**c**) PQ measured at various scan rates.

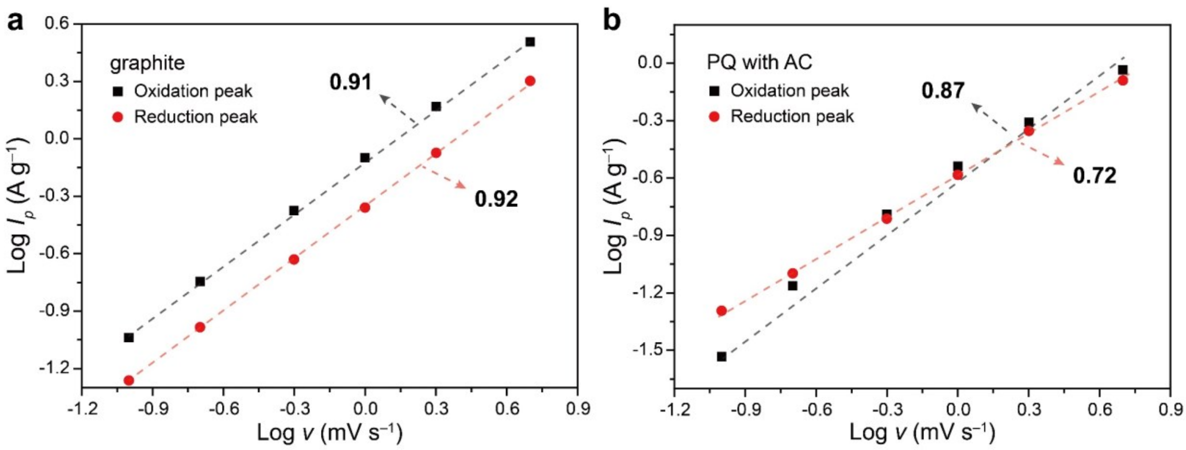

Supplementary Fig. 17 | log v-log I_p plots of (**a**) graphite and (**b**) PQ with b-values.

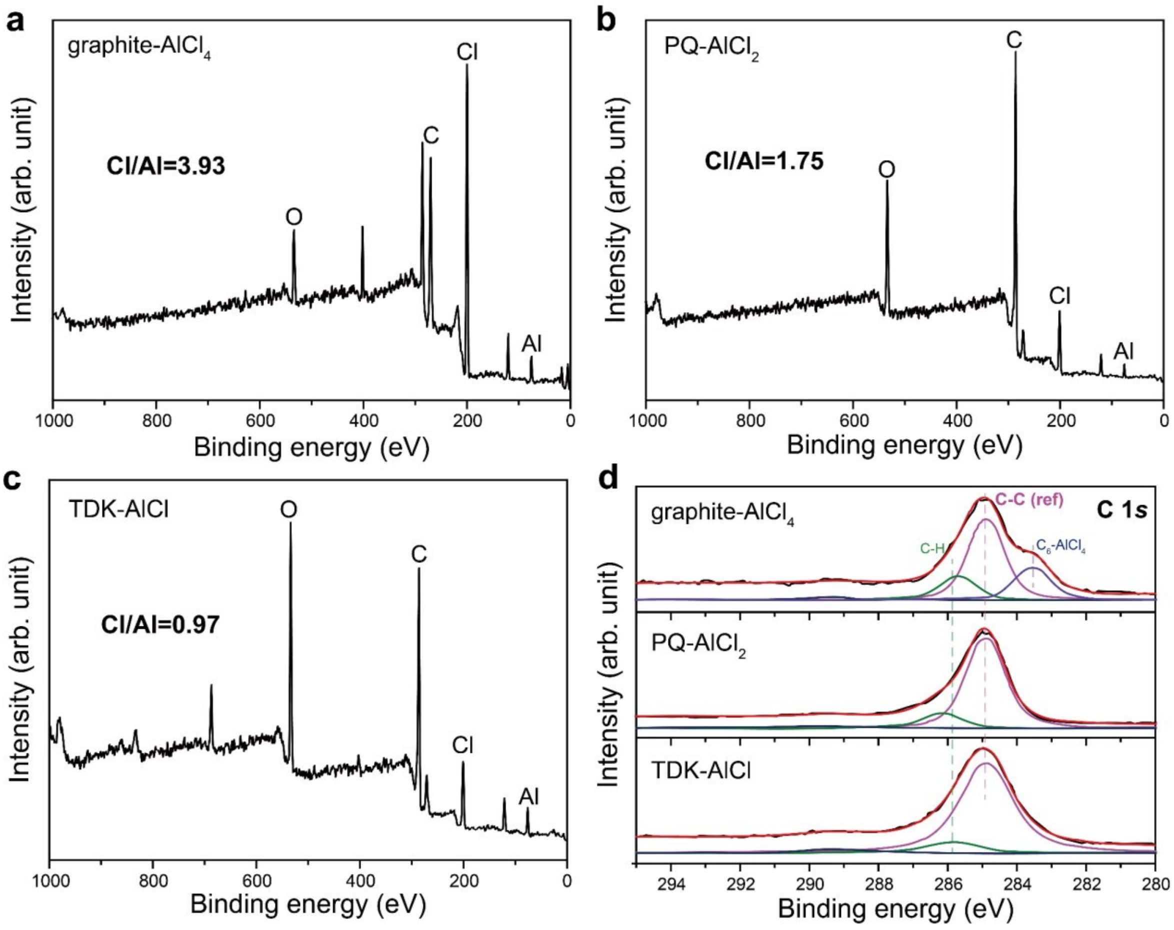

Supplementary Fig. 18 | Full range XPS profiles of (**a**) graphite-$AlCl_4$, (**b**) PQ-$AlCl_2$, and (**c**) TDK-AlCl, and (**d**) their XPS profiles in C 1*s* branches.

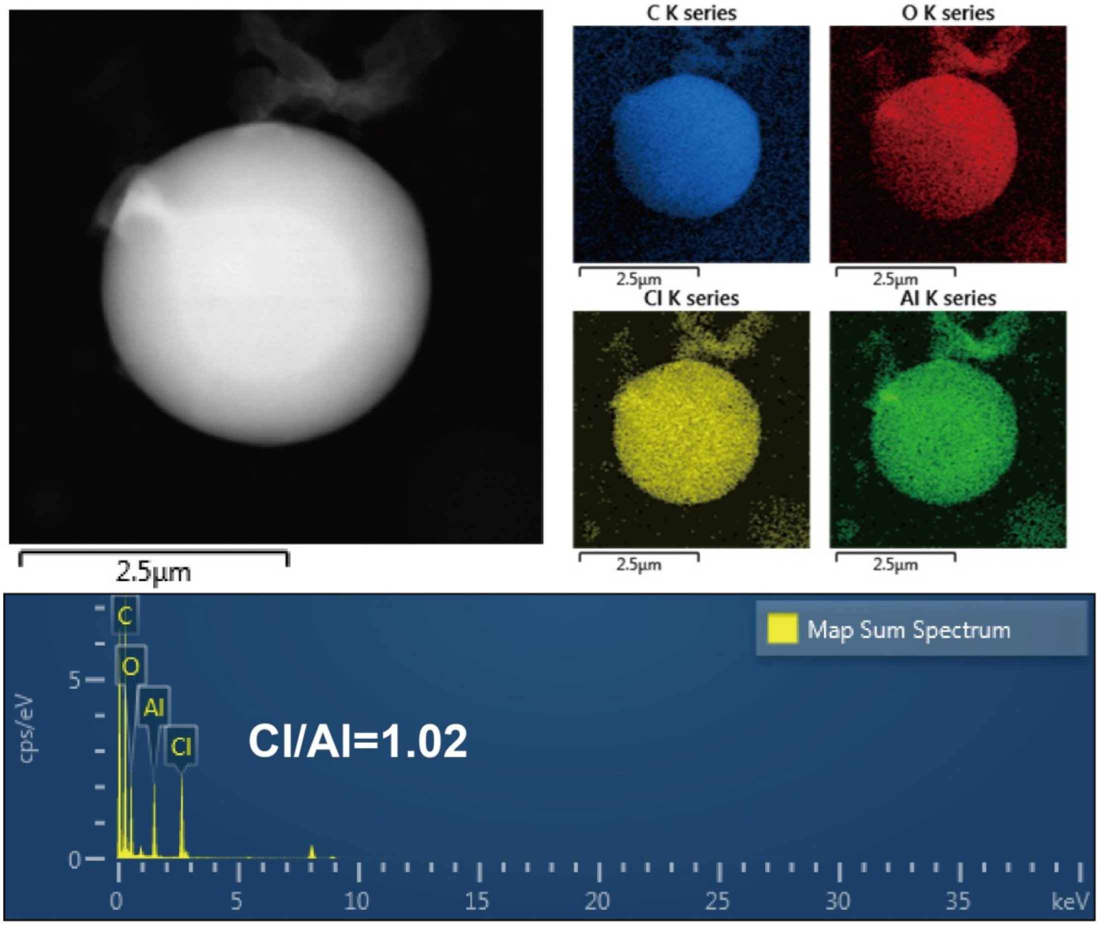

Supplementary Fig. 19 | TEM image and EDS mapping of discharged TDK-AC blend after 5 cycles.

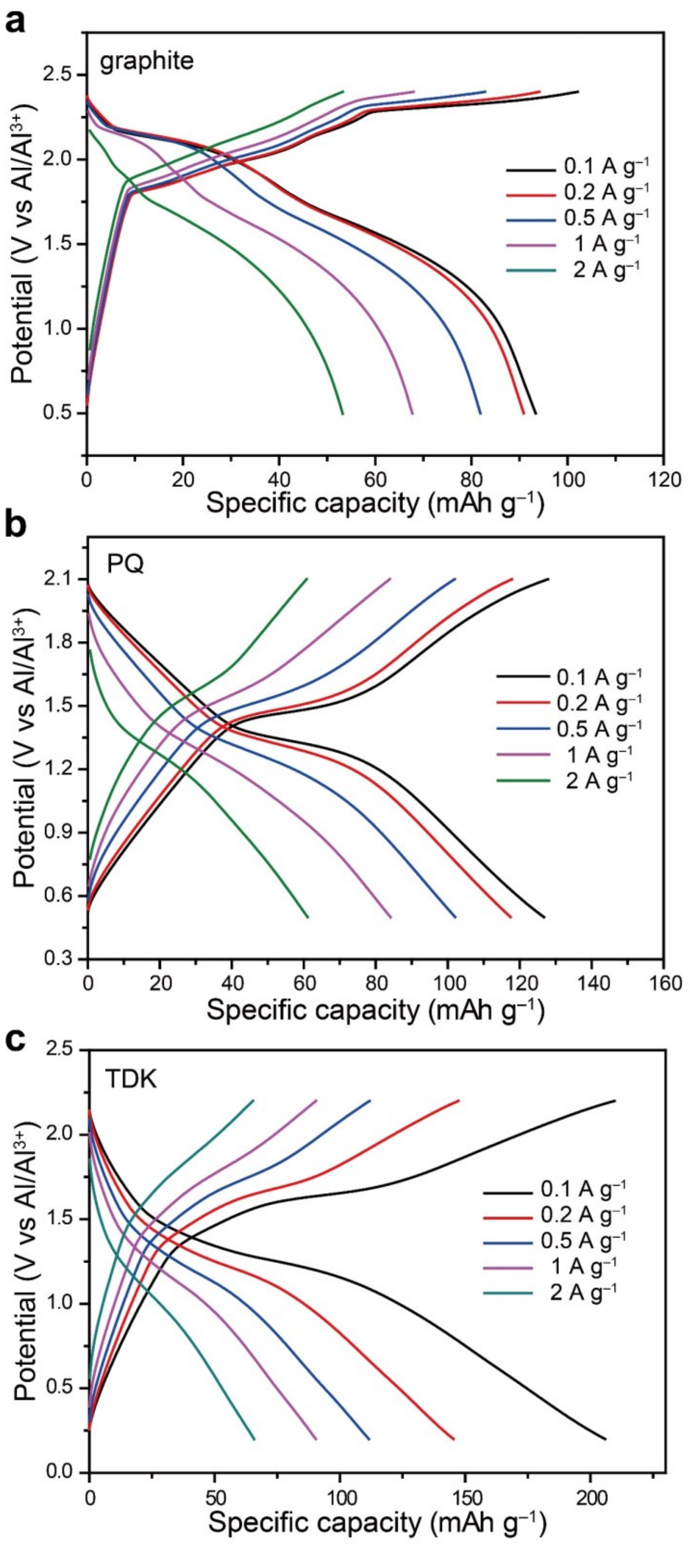

Supplementary Fig. 20 | Voltage profiles of (**a**) graphite, (**b**) PQ, and (**c**) TDK cells at various current densities.

Supplementary Note | Energy Density Calculation

Whereas Li-ion batteries operate under the mechanism known as "rocking chair" in which Li^+ ions only are exploited as carrier ions, aluminium ion batteries (AIBs) use different types of ions for the anode (Al^{3+}) and the cathode ($AlCl_4^-$, $AlCl_2^+$, or $AlCl^{2+}$). Therefore, a different formula is required for the energy density calculation of AIBs. The different types of carrier ions require a different amount of electrolyte to yield carrier ions with the designated compositions. Taking this into consideration, the following equation is established[1] to evaluate the total capacity that reflects the amount of electrolyte:

$$C_{total} = \frac{Fx(r-1)C_C}{Fx(r-1) + C_C(rM_{AlCl3} + M_{EMIMCl})}$$

,where F is the Faraday constant, 26.8×10^3 mAh mol^{-1}, x = number of electrons used to reduce 1 mole of anodic material (i.e., $AlCl_3$), r is the $AlCl_3$/[EMIm]Cl molar ratio, C_C is the specific capacity of the cathode (mAh g^{-1}), M_{AlCl3} is the molar mass of $AlCl_3$ (g mol^{-1}), and M_{EMIMCl} is the molar mass of [EMIm]Cl or any other source of Cl^- (g mol^{-1}).

A cell that uses graphite as the cathode material operates on the basis of the following reactions[2]:

$$\text{Anode: } \mathrm{AlCl_4^-} + \tfrac{1}{3}\mathrm{Al} \leftrightarrow \tfrac{4}{3}\mathrm{AlCl_3} + \mathrm{e^-}$$

$$\text{Cathode: } \mathrm{C_n[AlCl_4]} + \mathrm{e^-} \leftrightarrow \mathrm{C_n} + \mathrm{AlCl_4^-}$$

$$\text{Full-cell: } \mathrm{C_n[AlCl_4]} + \tfrac{1}{3}\mathrm{Al} \leftrightarrow \tfrac{4}{3}\mathrm{AlCl_3} + \mathrm{C_n} \quad (1\mathrm{e^-}\text{ reaction}),\ x = \frac{3}{4}$$

A cell that uses PQ as the cathode material operates on the basis of the following reactions[3]:

$$\text{Anode: } \mathrm{AlCl_4^-} + \tfrac{1}{3}\mathrm{Al} \leftrightarrow \tfrac{4}{3}\mathrm{AlCl_3} + \mathrm{e^-}$$

$$\text{Cathode: } \mathrm{PQ} + 2\mathrm{AlCl_3} + \mathrm{e^-} \leftrightarrow \mathrm{PQ[AlCl_2]} + \mathrm{AlCl_4^-}$$

$$\text{Full-cell: } \mathrm{PQ} + \tfrac{2}{3}\mathrm{AlCl_3} + \tfrac{1}{3}\mathrm{Al} \leftrightarrow \mathrm{PQ[AlCl_2]} \quad (1\mathrm{e^-}\text{ reaction}),\ x = \frac{3}{2}$$

A cell that uses TDK as the cathode material operates on the basis of the following reactions:

$$\text{Anode: } AlCl_4^- + \frac{1}{3}Al \leftrightarrow \frac{4}{3}AlCl_3 + e^-$$

$$\text{Cathode: } \frac{1}{8}TDK + \frac{3}{2}AlCl_3 + e^- \leftrightarrow \frac{1}{8}TDK[4AlCl] + AlCl_4^-$$

$$\text{Full-cell: } \frac{1}{8}TDK + \frac{1}{6}AlCl_3 + \frac{1}{3}Al \leftrightarrow \frac{1}{8}TDK[4AlCl] \quad (1e^- \text{ reaction}),\ x = 6$$

According to the above reactions, the x values for graphite, PQ, and TDK cathodes are calculated to be 3/4, 3/2, and 6 respectively, implying that TDK requires a much smaller amount of electrolyte for charge storage owing to the use of divalent Al-complex ions. The energy density is calculated by multiplication of the total capacity and operation voltage: $E = C_{total} \cdot V$. When these three different cathode materials are adopted, the following total capacities and energy densities are obtained:

Graphite cathode: C_{total} = 22.1 mA/g, Energy density = 38.7 Wh/kg (voltage = 1.75V)

PQ cathode: C_{total} = 39.7 mA/g, Energy density = 55.7 Wh/kg (voltage = 1.4V)

TDK cathode: C_{total} = 145.4 mA/g, Energy density = 189 Wh/kg (voltage = 1.3V)

Supplementary References

1 Kravchyk, K. V., Wang, S., Piveteau, L. & Kovalenko, M. V. Efficient Aluminum Chloride–Natural Graphite Battery. *Chem. Mater.* **29**, 4484-4492 (2017).

2 Lin, M.-C. *et al.* An ultrafast rechargeable aluminium-ion battery. *Nature* **520**, 324-328 (2015).

3 Kim, D. J. *et al.* Rechargeable aluminium organic batteries. *Nat. Energy* **4**, 51-59 (2019).
