# Supplementary material for: Tetradiketone macrocycle for divalent aluminium ion batteries
Source: Nat Commun. 2021 Apr 22;12:2386. doi: 10.1038/s41467-021-22633-y (PMC8062564; doi:10.1038/s41467-021-22633-y)
Supplement: Supplementary file 2 — Description of Additional Supplementary Files [file 41467_2021_22633_MOESM2_ESM.docx]

**Description of Additional Supplementary Files**

File Name: Supplementary video 1

Description: The C=O asymmetric stretching modes of TDK at 1686 cm^‒1^.

File Name: Supplementary video 2

Description: The C–C and C=C stretching modes near the C=O at 1581 cm^‒1^ for TDK-1AlCl.

File Name: Supplementary video 3

Description: The C–C and C=C stretching modes near the C=O at 1527 cm^‒1^ for TDK-1AlCl.

File Name: Supplementary video 4

Description: The C–C and C=C stretching modes of TDK at 1180 cm^‒1^.

File Name: Supplementary video 5

Description: The C–C and C=C stretching modes of TDK-4AlCl at 1210 cm^‒1^.

File Name: Supplementary video 6

Description: The C–C and C=C stretching modes and C=O stretching modes at 1013 cm^‒1^.

File Name: Supplementary video 7

Description: The C–C and C=C stretching modes and C=O stretching modes of TDK-4AlCl at 1013 cm^‒1^.

File Name: Supplementary video 8

Description: The C–H wagging and C=O asymmetric stretching modes of TDK at 880 cm^‒1^.

File Name: Supplementary video 9

Description: The C–H wagging and O–Al stretching modes of TDK-4AlCl at 844 cm^‒1^.
